# Liver Ischemic Preconditioning (IPC) Improves Intestinal Microbiota Following Liver Transplantation in Rats through 16s rDNA-Based Analysis of Microbial Structure Shift

**DOI:** 10.1371/journal.pone.0075950

**Published:** 2013-10-02

**Authors:** Zhigang Ren, Guangying Cui, Haifeng Lu, Xinhua Chen, Jianwen Jiang, Hui Liu, Yong He, Songming Ding, Zhenhua Hu, Weilin Wang, Shusen Zheng

**Affiliations:** 1 Key Laboratory of Combined Multi-organ Transplantation, Ministry of Public Health, First Affiliated Hospital, School of Medicine, Zhejiang University, Hangzhou, China; 2 Department of Hepatobiliary and Pancreatic Surgery, First Affiliated Hospital, School of Medicine, Zhejiang University, Hangzhou, China; 3 State Key Laboratory for Diagnosis and Treatment of Infectious Disease, First Affiliated Hospital, School of Medicine, Zhejiang University, Hangzhou, China; 4 Institute of Immunology, Zhejiang University School of Medicine, Hangzhou, China; UNIFESP Federal University of São Paulo, Brazil

## Abstract

**Background:**

Ischemia-reperfusion (I/R) injury is associated with intestinal microbial dysbiosis. The “gut-liver axis” closely links gut function and liver function in health and disease. Ischemic preconditioning (IPC) has been proven to reduce I/R injury in the surgery. This study aims to explore the effect of IPC on intestinal microbiota and to analyze characteristics of microbial structure shift following liver transplantation (LT).

**Methods:**

The LT animal models of liver and gut IPC were established. Hepatic graft function was assessed by histology and serum ALT/AST. Intestinal barrier function was evaluated by mucosal ultrastructure, serum endotoxin, bacterial translocation, fecal sIgA content and serum TNF-α. Intestinal bacterial populations were determined by quantitative PCR. Microbial composition was characterized by DGGE and specific bacterial species were determined by sequence analysis.

**Principal Findings:**

Liver IPC improved hepatic graft function expressed as ameliorated graft structure and reduced ALT/AST levels. After administration of liver IPC, intestinal mucosal ultrastructure improved, serum endotoxin and bacterial translocation mildly decreased, fecal sIgA content increased, and serum TNF-α decreased. Moreover, liver IPC promoted microbial restorations mainly through restoring *Bifidobacterium* spp., Clostridium clusters XI and Clostridium cluster XIVab on bacterial genus level. DGGE profiles indicated that liver IPC increased microbial diversity and species richness, and cluster analysis demonstrated that microbial structures were similar and clustered together between the NC group and Liver-IPC group. Furthermore, the phylogenetic tree of band sequences showed key bacteria corresponding to 10 key band classes of microbial structure shift induced by liver IPC, most of which were assigned to Bacteroidetes phylum.

**Conclusion:**

Liver IPC cannot only improve hepatic graft function and intestinal barrier function, but also promote restorations of intestinal microbiota following LT, which may further benefit hepatic graft by positive feedback of the “gut-liver axis”.

## Introduction

Currently, liver transplantation (LT) has been accepted as an established therapy for various end-stage liver diseases for more than three decades [1,2]. Nowadays, the survival rate of patients following LT approximately is 90% for 1 year and 75% for 5 years [3]. During LT, the recipients inevitably suffer from the dual ischemia-reperfusion (I/R) injuries from liver and intestine. These injuries are closely associated with many postoperative complications, and they likely lead to endogenous infections, increasing postoperative mortality rate [4].

In recent years, intestinal microbiota has been considered as the most important micro-ecosystem and equivalent to a major metabolic "organ" that has a symbiotic relationship with the body [5–7]. The human intestine accommodates nearly 400 different species of bacteria and comprises 10^13^ to 10^14^ microorganisms whose collective genome contains at least 100 times as large as human beings genome [5,8]. The vast majority of these microbes (10 to 100 trillion) inhabit human gastrointestinal tract, play a crucial role on human physiology and nutrition, and are essential for human survival and life [9,10]. Current evidences have demonstrated that intestinal I/R injury in surgery or intestinal transplantation would result in colonic flora dysbiosis that follows epithelia damage, which is mainly derived from overgrowth of genera *Escherichia coli* and *Prevotella oralis* [11,12], Also, hepatic I/R injury can lead to imbalance of intestinal microbiota, expressed by the increased *Enterobacteria* as well as the decreased *Lactobacilli* and *Bifidobacteria* [13].

The “gut-liver axis” closely links gut function and liver function in health and disease. Under disease situation, intestinal microbiota imbalance can aggravate liver injury and promote chronic inflammatory disease of the liver [14]. Meanwhile, hepatic injury or disease, such as nonalcoholic steatohepatitis, alcoholic steatohepatitis, and cirrhosis, always follows changes in intestinal permeability and microbial composition [14].

Ischemic preconditioning (IPC), defined as brief periods of ischemia and reperfusion before sustained ischemia, has been shown a protective role in several organs that results in an increased tolerance towards organ hypoxia [15–17]. This method was firstly reported by Murry et al [18] in a study on dog heart tissues, and it has since been proven to significantly reduce I/R-injury in experimental and clinical researches [15,16,19]. However, until now, we have not found the relative reports on the effect of IPC on intestinal microbiota and microbial change characteristics after IPC in organ transplantation.

In this study, we established the LT model of IPC and undertook the study (1) to observe the effect of IPC on hepatic graft function and intestinal barrier function, and (2) to characterize microbial structure shift related to IPC following LT in rats using denaturing gradient gel electrophoresis (DGGE) method.

## Materials and Methods

### Experimental design

#### Experimental protocol

**Figure 1:**
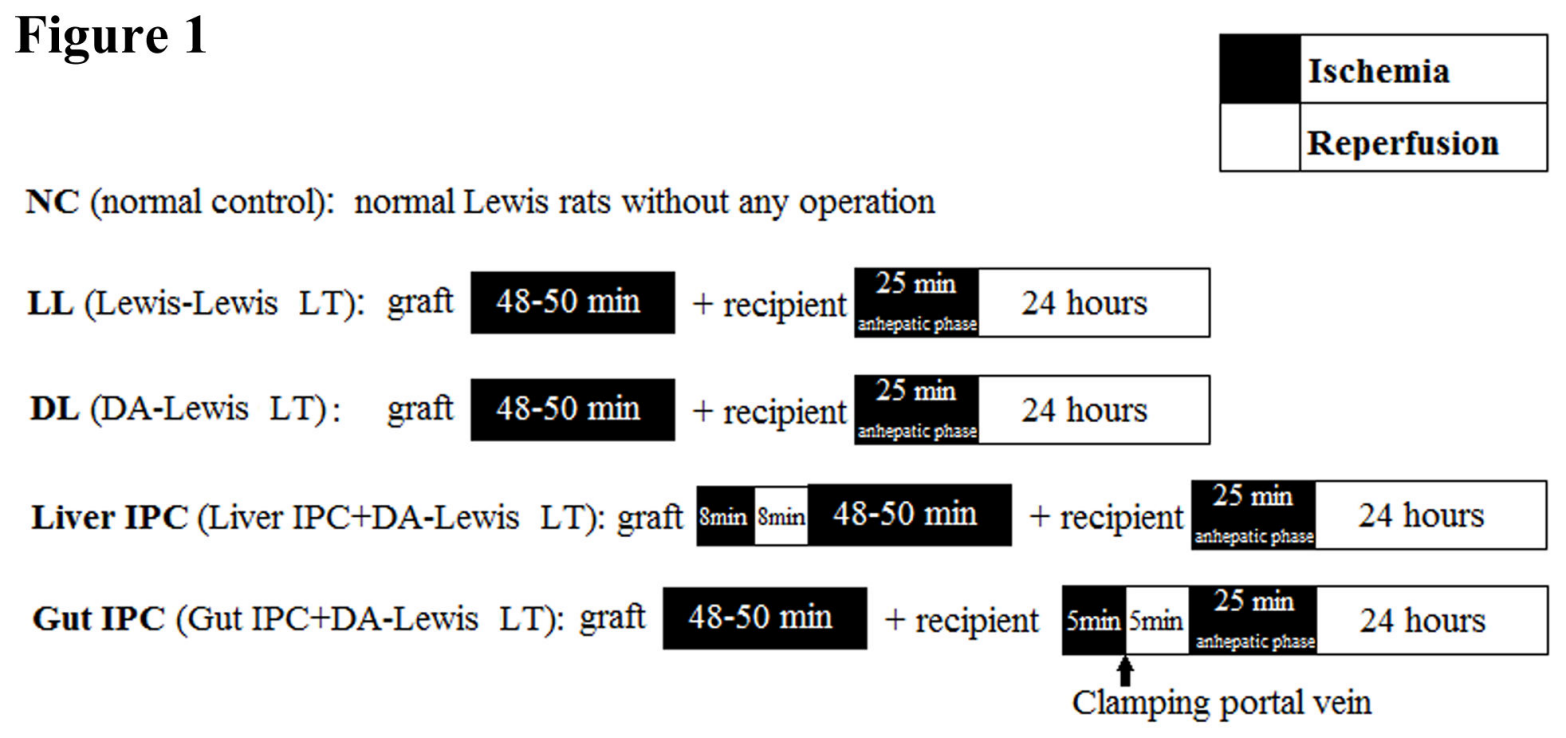
Experimental protocol.

During the operation of LT in rats, the donor liver inevitably suffered from 48-50 min ischemic injury, and the recipient also underwent about 25 min anhepatic phase, so we designed the following IPC experimental protocol as shown in Figure 1. The whole experiment was divided into 5 groups. (i) NC group (normal control, n=8): normal Lewis rats without any operation (ii). LL group (Lewis-Lewis LT, n=6): both donors and recipients were Lewis rats (iii). DL group (DA-Lewis LT, n=6): donors were DA rats and recipients were Lewis rats (iv). Liver IPC group (Liver IPC+DA-Lewis LT, n=6): donor liver received 8 min ischemia and subsequently 8 min reperfusion by clamping (ischemia) and declamping (reperfusion) hepatic artery and portal vein before the graft was removed, and then LT operation was performed. (v) Gut IPC group (Gut IPC+DA-Lewis LT, n=6): recipient’s gut received 5 min blood stasis and subsequently 5 min blood flow by clamping (blood stasis) and declamping (blood flow) portal vein before the anhapatic phase of LT. All procedures were performed according to the ‘‘Guide for the Care and Use of Laboratory Animals’’ published by the National Institutes of Health (NIH publication 86–23 revised 1985). And the protocols were approved by Animal Care and Use Committee of the First Affiliated Hospital, School of Medicine, Zhejiang University.

#### Surgical procedures

Rats were anesthetized with Ketamine Hydrochloride (100 mg/kg intraperitoneal). The LT models of rats were established according to previous techniques [20,21], with slight modifications. Briefly, after the donor liver was dissociated, the graft was perfused with chilled saline containing 25U/mL heparin through the portal vein, and then preserved in cold normal saline. After the liver of the recipient was removed, the graft was placed into the recipient’s abdomen. After the anastomosis of suprahepatic vene cava and portal vein of the recipient was finished, the hepatic graft was reperfused. The common bile duct was reconstructed by tying the duct over a stent. All recipients recovered in a short time, and no further treatment was performed.

#### Sample collection

All recipient rats were sampled after 24 h of reperfusion. The abdominal aorta was punctured and blood samples were collected for measurements of serum endotoxin, serum TNF-α and liver enzymes. Hepatic graft samples and intestinal tissues were harvested for further analysis. Rats were then sacrificed by over-dose anesthesia. The ileocecal contents were collected and immersed in liquid nitrogen, and then stored at -80°C for further analysis of intestinal microbiota.

### Microbial analysis of the ileocecal content

#### Quantitative PCR (qPCR) to detect bacterial population

The primers for the genetic determinants were used in earlier studies (Table **S1**).

### Statistical analysis

All data are expressed as mean ± standard error (SEM). Statistical analyses were performed with the software package SPSS for Windows (version 17.0; SPSS, Inc., Chicago, IL, USA). For parametric data, statistical significance among groups was analyzed by one-way ANOVA analysis followed by post hoc Bonferroni’s multiple comparison tests. For non-parametric data, Kruskal-Wallis followed by Dunn’s multiple comparison tests was used. Statistical significance was set at *p* < 0.05.

For the other detailed methods see Text S1.

## Results

### Liver IPC improved hepatic graft function following LT in rats

**Figure 2:**
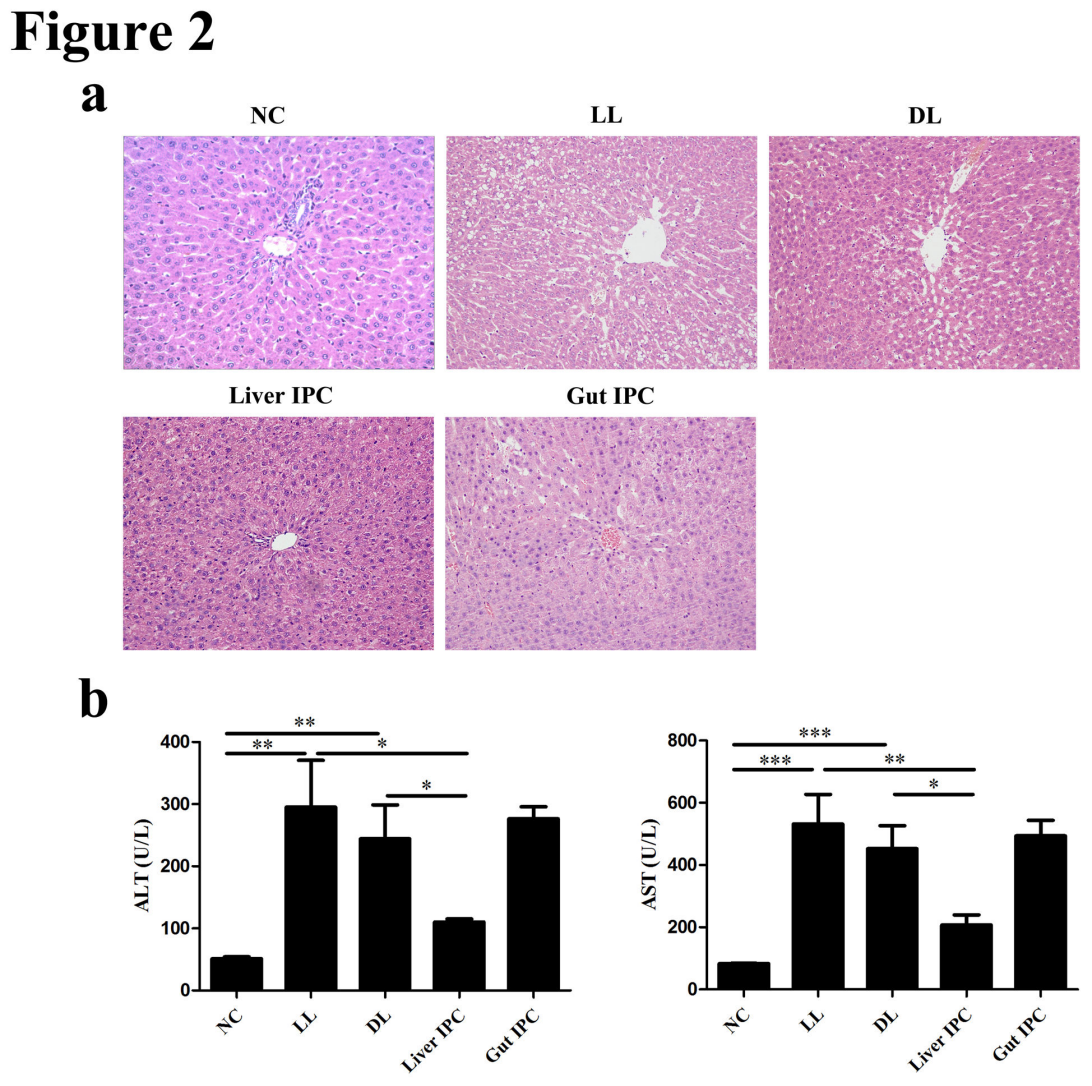
Liver IPC improved hepatic graft function following LT in rats. (a) Representative hepatic graft histopathology stained with hematoxylin and eosin (H&E, 200×). (b) Changes of liver enzymes (ALT and AST) in the serum of rats following LT.

To address the effect of IPC in graft I/R injury during LT, we observed graft histopathology and detected liver functions (ALT and AST), as shown in Figure 2. The severity of hepatic graft I/R injury was evaluated by Suzuki’s criteria [22,23]. Under light microscope in Figure 2a, the liver in the NC group showed normal structure with well-arranged hepatocyte cords (Suzuki’s score, 0). Both the LL and DL groups showed disarray of hepatocyte cords, vacuolization, local hepatocellular degeneration and necrosis, and widened sinusoids with inflammatory cell infiltration (Suzuki’s score, 4.17±0.40 and 3.83±0.51, respectively). Hepatic graft injuries were significantly ameliorated in the Liver IPC group (score, 1.33±0.21) compared to the DL group (*p* < 0.001). But hepatic graft histopathology had little improvement in the Gut IPC group (score, 3.67±0.33) versus the DL group.

As shown in Figure 2b, serum ALT and AST were remarkably increased in the LL group (295.0±75.52 U/L and 531.7±95.21 U/L, respectively) and the DL group (244.2±54.58 U/L and 452.5±73.55 U/L, respectively) compared to the NC group (51.00±3.27 U/L and 83.00±1.00, respectively, *p* < 0.01 or 0.001). Liver IPC profoundly reduced serum levels of ALT and AST (110.00±5.16 U/L and 206.7±32.88, respectively, both *p* < 0.05), compared to the DL group. But no difference in liver enzymes was noted in the Gut IPC group versus the DL group.

These results indicated that liver IPC could reduce hepatic graft I/R injury, while gut IPC had little improvement in hepatic I/R injury following LT in rats.

### Liver IPC improved intestinal barrier function following LT in rats

**Figure 3:**
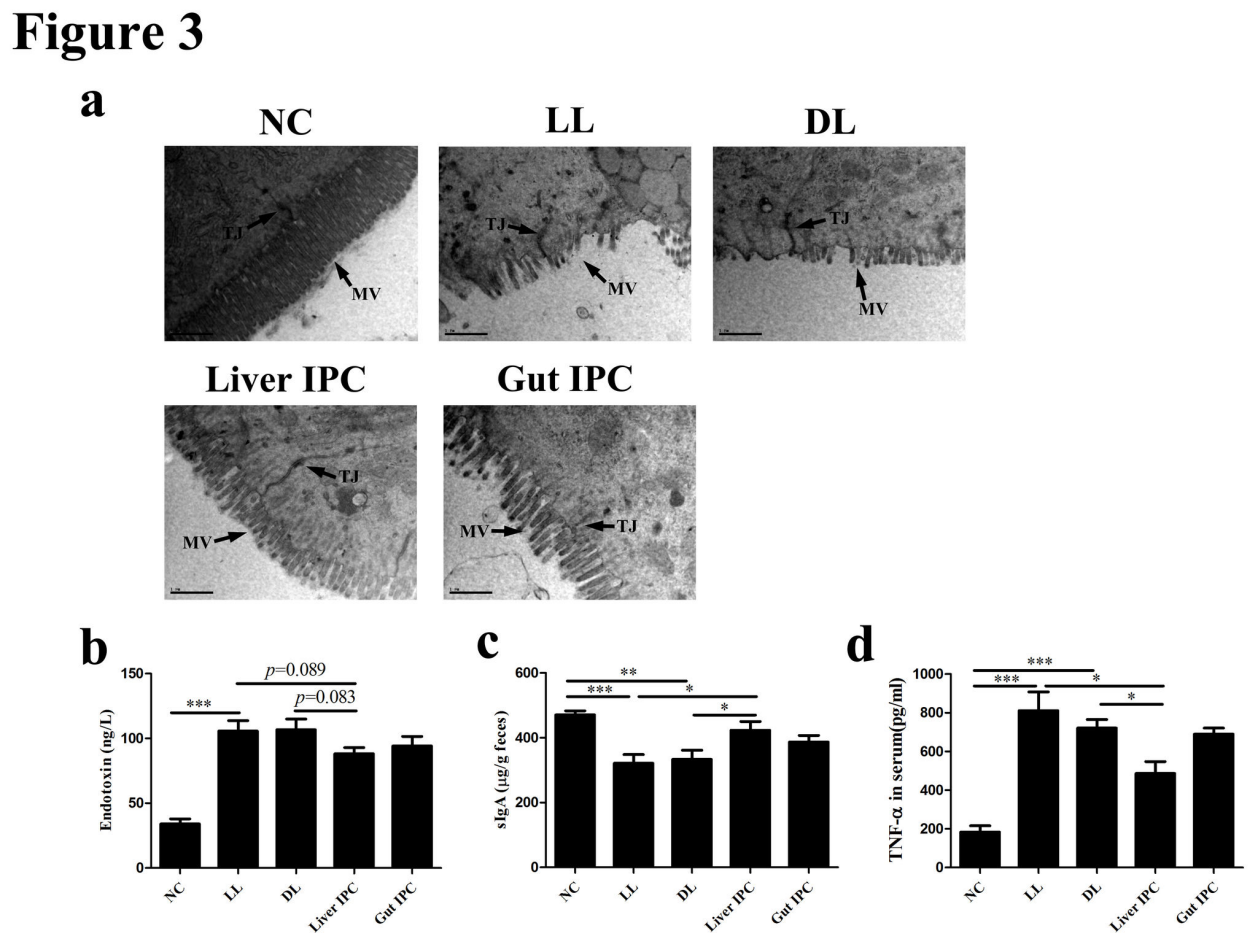
Liver IPC improved intestinal barrier function and inflammation following LT in rats. (a) Representative intestinal mucosal ultrastructure obtained by transmission electron microscopy (TEM) in rats following LT. MV: microvilli, TJ: tight junction. (b) Serum endotoxin was detected in the different groups. (c) The content of sIgA in wet feces was calculated by ELISA and expressed as nanogram (ng) per gram wet feces in the different groups. (d) Serum TNF-α was determined by ELISA in the different groups. *p<0.05, **p<0.01, ***p<0.001.

To evaluate the influence of IPC on intestinal mucosal integrity, we observed the ultrastructure of ileal mucosa by TEM shown in Figure 3a. In the NC group, intestinal epithelial cells showed normal ultrastructure with many homogenously distributed microvilli. Lateral junction complexes and desmosomes were also noted. Intestinal epithelial integrity in the LL and DL groups were damaged, evidenced by microvilli disruption and loss, destroyed tight junction and the wider lateral spaces between neighboring cells. In contrast, intestinal mucosal ultrastructures were significantly ameliorated in the Liver IPC group and mildly improved in the Gut IPC group compared to the DL group.

To test the effect of IPC on endotoxemia and bacterial translocation, serum endotoxin levels were determined and bacterial culture in the blood was performed. Rats from the LL and DL groups showed remarkably higher endotoxin levels (105.70±7.99 ng/L and 106.70±8.33 ng/L, respectively) than those from the NC group (34.00±3.90 ng/L, both *p* < 0.001). Notably, compared with the DL group, liver IPC mildly decreased endotoxin levels (88.17±4.77 ng/L), although there was no statistical difference (*p*=0.083), as shown in Figure 3b. Moreover, the positive rates of bacterial culture in the blood were elevated in the LL group (4/6, 66.7%) and DL group (3/6, 50%) versus the NC group (0%). A mild decrease in the Liver IPC group was also noted (2/6, 33.3%), but Gut IPC group still showed a high positive rate (3/6, 50%) (Table **S2**).

To determine the role of IPC on intestinal immune barrier function and inflammation, we detected fecal sIgA content and serum TNF-α level. Fecal sIgA contents were obviously lower in both the LL and DL groups (321.20±26.91 µg/g and 333.20±28.41 µg/g, respectively) than the NC group (471.00±12.28 µg/g, *p* < 0.001 and 0.01), but an obvious increase was noted in the Liver IPC group (423.10±27.03 µg/g, *p* < 0.05) versus the DL group (Figure 3c). Gut IPC also mildly elevated the content of fecal sIgA (386.88±20.62 µg/g). As shown in Figure 3d, the concentrations of serum TNF-α were significantly increased in both the LL and DL groups (811.10±96.75 pg/ml and 721.20±44.10 pg/ml, respectively) compared to the NC group (183.80±32.18 pg/ml, both *p* < 0.001). Contrastingly, the concentration had a remarkable decrease in the Liver IPC group (486.60±61.51 pg/ml) compared with the DL group (*p* < 0.05). But gut IPC had no influence on the concentration of serum TNF-α.

These results demonstrated that liver IPC could improve intestinal barrier function and inflammation, while gut IPC only improved mildly intestinal mucosal integrity following LT in rats.

### Quantitative analysis of predominant bacteria in feces by qPCR

**Figure 4:**
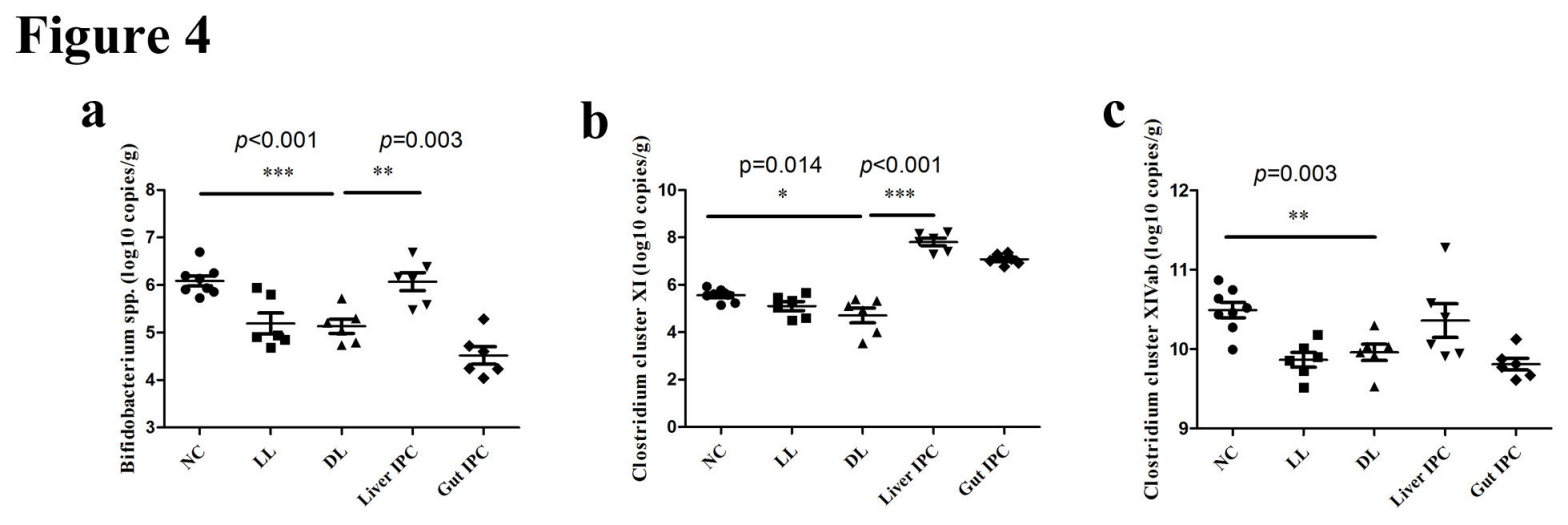
Quantitative analysis of characteristic bacteria in feces. Log_10_ copies/g: log_10_ no. of 16S rDNA gene copies per gram feces (wet weight). On the level of bacterial genus, specific bacterial populations were determined by real-time qPCR in the different groups: (a) *Bifidobacterium* spp., (b) Clostridium cluster XIVab and (c) Clostridium clusters XI. Comparisons of specific bacteria among the NC group, DL group and Liver-IPC group were emphasized. Statistical differences were calculated by one-way ANOVA. *p<0.05, **p<0.01, ***p<0.001.

To determine the influence of IPC on intestinal microflora following LT in rats, 10 predominant bacteria on the level of bacterial genus were analyzed by qPCR. Bacterial amounts in the different groups were shown in Table **1**, and the characteristic bacterial quantities were shown in Figure 4. The numbers of 16S rDNA gene copies of *Bifidobacterium* spp. and Clostridium clusters XI were remarkably decreased in the DL group versus NC group (*p* < 0.001 and *p* = 0.014, respectively), but restored to the normal level in the Liver IPC group versus the DL group (*p* = 0.003 and *p* < 0.001, respectively) (Figure 4 a& b). And the number of Clostridium cluster XIVab also had this restored trend (Figure 4 c). The numbers of Enterobacteriaceae, *Enterococcus* and *Lactobacillus* almost had no significant differences among the different groups. Moreover, the numbers of *Bacteroides* and *Faecalibacterium prausnitzii* were decreased in the LL, DL, Liver IPC and Gut IPC groups versus the NC group (Gut IPC group data not shown). The numbers of *Clostridium bolteae* and Clostridium clusters I had no obvious changes between the NC group and the LL/DL groups, and became decreased in the Liver IPC and Gut IPC groups.

**Table 1:** Quantity of predominant bacteria in feces by real-time qPCR.

Bacterial genera	*Bacterial counts (copies/g)#*					*p value one-way ANOVA*	
	NC	LL	DL	Liver-IPC		NC vs. DL	DL vs. Liver-IPC
*Bacteroides*	10.25±0.14	8.44±0.17	8.01±0.23	8.28±0.19		<0.001***	0.388
*Bifidobacterium* spp.	6.09±0.11	5.19±0.22	5.13±0.15	6.07±0.19		<0.001***	0.003**
*Clostridium* cluster I	7.88±0.13	7.35±0.09	7.88±0.15	6.53±0.13		0.994	<0.001***
*Clostridium* cluster XI	5.56±0.09	5.10±0.19	4.71±0.31	7.81±0.16		0.014*	<0.001***
*Clostridium* cluster XIVab	10.49±0.10	9.87±0.09	9.96±0.10	10.36±0.21		0.003**	0.121
*Clostridium bolteae*	4.30±0.16	3.63±0.29	3.88±0.11	1.83±0.31		0.062	<0.001***
*Faecalibacterium prausnitzii*	9.00±0.10	8.24±0.11	8.28±0.08	8.26±0.08		<0.001***	0.868
Lactobacillus	9.54±0.20	9.19±0.36	9.85±0.16	8.64±0.35		0.273	0.011*
Enterococcus	8.07±0.23	7.93±0.10	8.05±0.14	7.23±0.33		0.946	0.057
Enterobacteriaceae	8.40±0.36	8.05±0.30	8.00±0.28	7.13±0.20		0.419	0.030*

# log_10_ no. of 16S rDNA gene copies per gram (wet weight). Values were shown as mean±standard errors (SE). Statistical differences were calculated by one-way ANOVA. Comparisons of predominant bacteria among the NC group, DL group and Liver-IPC group were emphasized. *p<0.05, **p<0.01, ***p<0.001.

These results suggested that liver IPC promoted intestinal microbial restoration mainly through restoring genera *Bifidobacterium*, Clostridium clusters XI and Clostridium cluster XIVab on the level of bacterial genus following LT in rats.

### Liver IPC improved intestinal microbiota following LT in rats shown in DGGE profiles

#### Intestinal microbial diversity analysis

**Figure 5:**
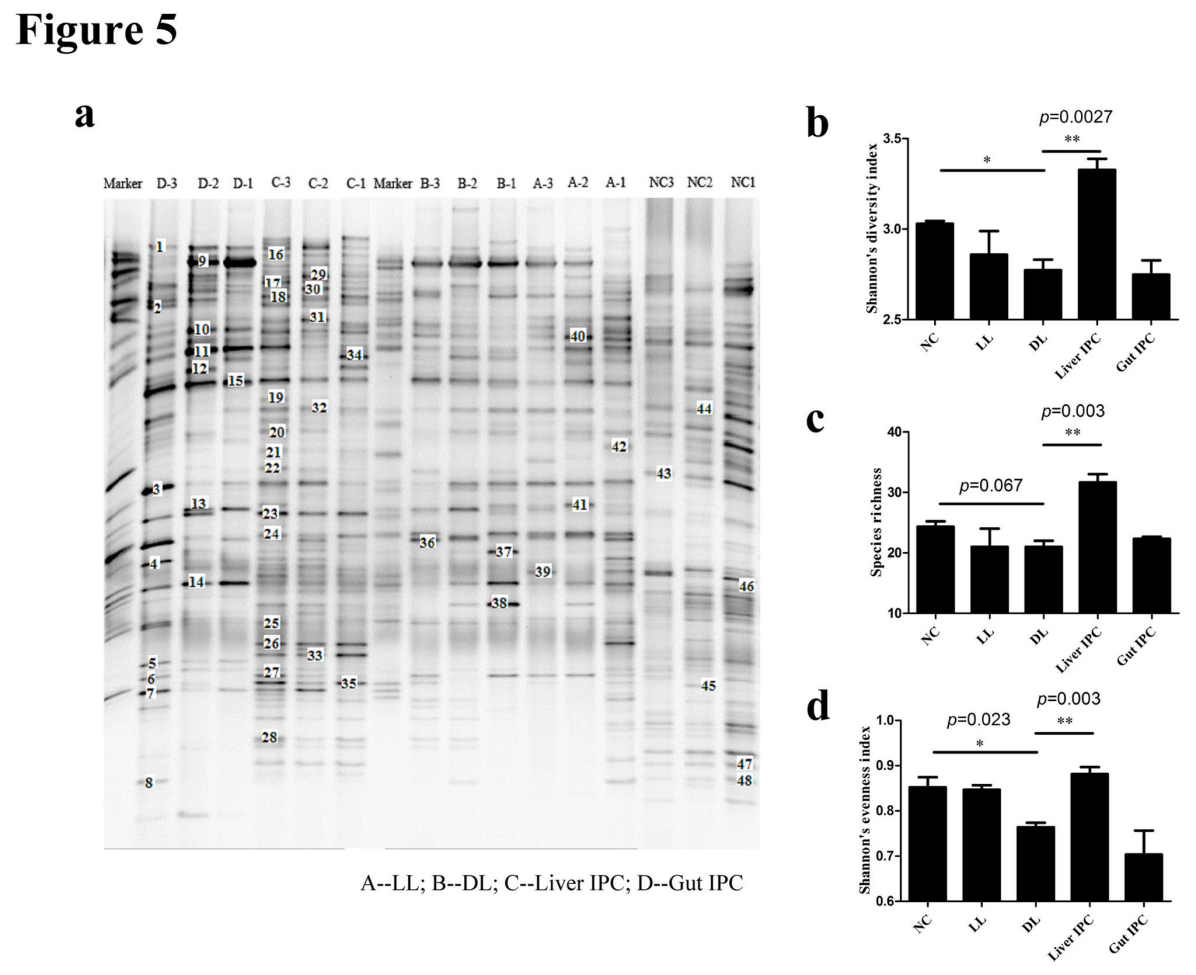
Liver IPC improved intestinal microbiota following LT in rats shown in DGGE profiles. (a) DGGE profiles of fecal bacteria in rats from the different groups. Sample numbers above lanes indicated the different rats from the different groups. Marker lane was used for gel to gel comparison. Each band represents a bacterial clone. Band numbers (corresponding to Figure 7 band classes) indicated the position of bands excised for sequence analyses (e.g. ‘“20”’ means band 20). (b) Intestinal microbial diversity comparison (Shannon’s diversity index). (c) Species richness comparison. (d) Shannon’s evenness index comparison. *p<0.05, **p<0.01.

The DGGE profiles of ileocecal flora showed shifts of intestinal microbial community composition (Figure 5a). The changes of DGGE band profiles in the different groups were difficult to be quantified only by observation, so we first calculated the gray amount of each band of each lane in the DGGE profiles with Gel-Pro analyzer software, and then analyzed the microbial diversity using the Past software. As shown in Figure 5b, Shannon’s diversity index was decreased in the DL group versus the NC group (2.77±0.06 vs. 3.03±0.01, *p* < 0.05), and increased in the Liver IPC group (3.33±0.06) versus the DL group (*p* = 0.0027). Meanwhile, the species richness was mildly low in the DL group compared to the NC group (21.00±1.00 vs. 24.33±0.88, *p* = 0.067), and increased in the Liver IPC group (31.67±1.33) versus the DL group (*p* = 0.003) shown in Figure 5c. Moreover, Shannon’s evenness index was lower in the DL group than the NC group (0.76±0.01 vs. 0.85±0.02, *p* = 0.023), and significantly higher in the Liver IPC group (0.88±0.02) than the DL group (*p* = 0.003) (Figure 5d). But no remarkable changes were noted between the Gut IPC group and the LL/DL groups in these diversity indexes.

These results indicated that liver IPC could increase intestinal microbial diversity and species richness following LT in rats.

#### Cluster analysis of DGGE profiles

**Figure 6:**
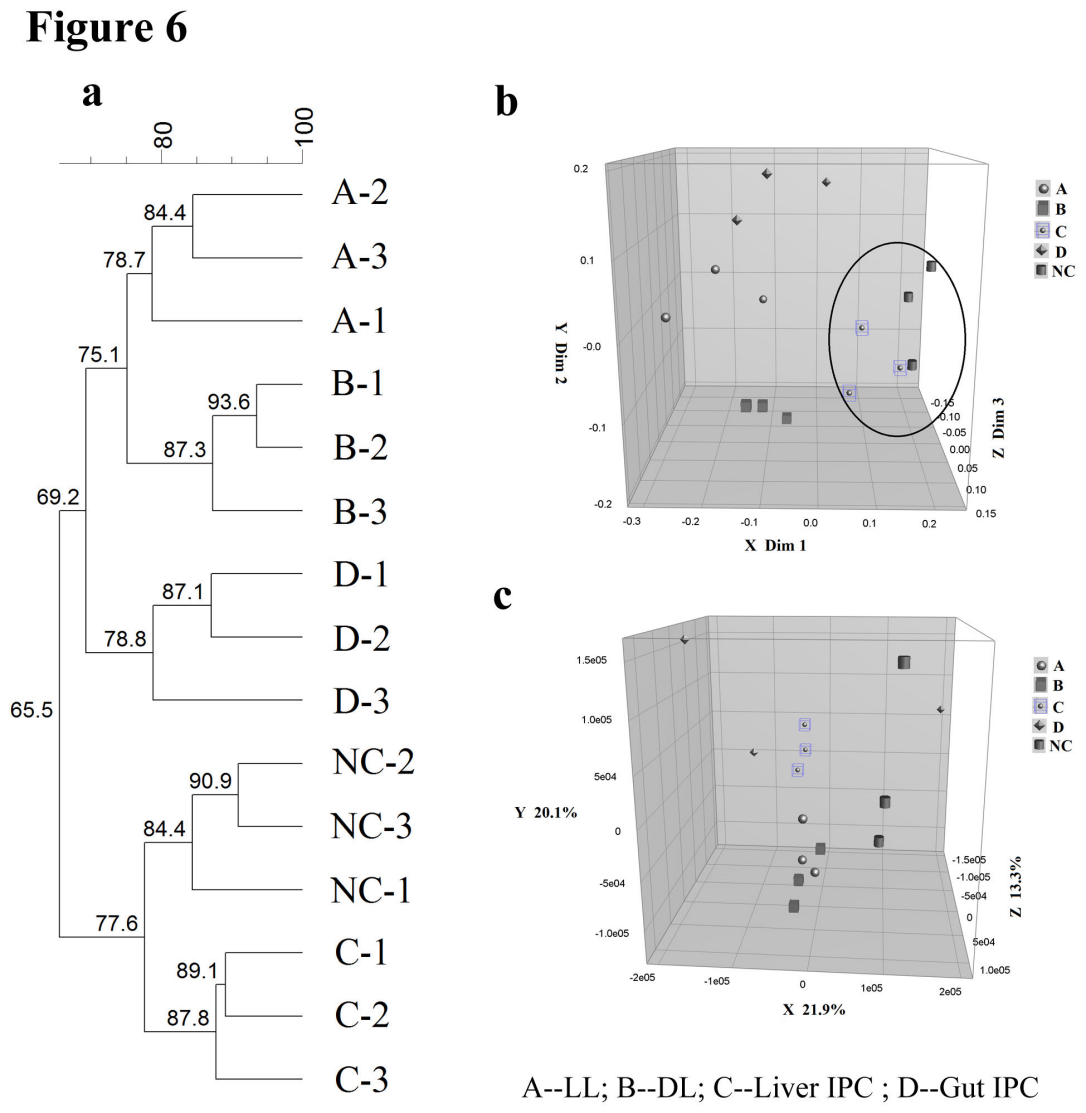
Cluster analysis of DGGE profiles obtained with universal primers V3 using Dice’s coefficient and UPGMA. (a) Cluster analysis of DGGE profiles from the different groups. Metric scale denotes the degree of similarity. (b) Multidimensional scaling (MDS) analysis of the cluster shown in (a). The plot is an optimized three-dimensional representation of the similarity matrix obtained from BioNumerics software, and the *x*-, *y*-, and *z*-axes separately represent three different dimension: Dim 1, Dim 2, and Dim 3. Euclidean distance between two points reflects similarity. (c) Principal components analysis (PCA) of fecal microbiota based on DGGE fingerprinting shown in (a). It reorients the plot to maximize the variation among lanes along the first three principal components (the contributions 21.9, 20.1 and 13.3, respectively) obtained from BioNumerics software.

To analyze characteristics of DGGE profiles from the different groups, we utilized the Dice coefficient and UPGMA as a cluster method to indicate band pattern similarity (Figure 6). As shown in Figure 6a, these profiles formed two primary clusters. The above cluster contained the samples from the LL, DL and Gut-IPC groups, whereas the below one contained the samples from the NC and Liver-IPC groups. The total similarity of the first cluster was 69.2%, while the second cluster was 77.6%. Moreover, the similarity of the lanes from each group ranged from 78.7% to 93.6%, which suggested a uniqueness and stability of samples from each group.

In addition, these change characteristics of DGGE profiles among the different groups were also confirmed by MDS (Figure 6b) and PCA (Figure 6c). The distance between 2 data points represents the extent of difference between the 2 rats’ gut microbial compositions. Microbial structures of rats from the NC group and the Liver-IPC group were clustered together, and showed a separation from the other groups by MDS (Dim 1, Dim 2, and Dim 3) and PCA axis X/Y/Z (contribution rate: 21.9%, 20.1 and 13.3%, respectively).

These results demonstrated that intestinal microbial structures in rats from the NC group and Liver-IPC group were similar and clustered together.

#### Phylogenetic tree analysis of sequences from DGGE profiles

**Figure 7:**
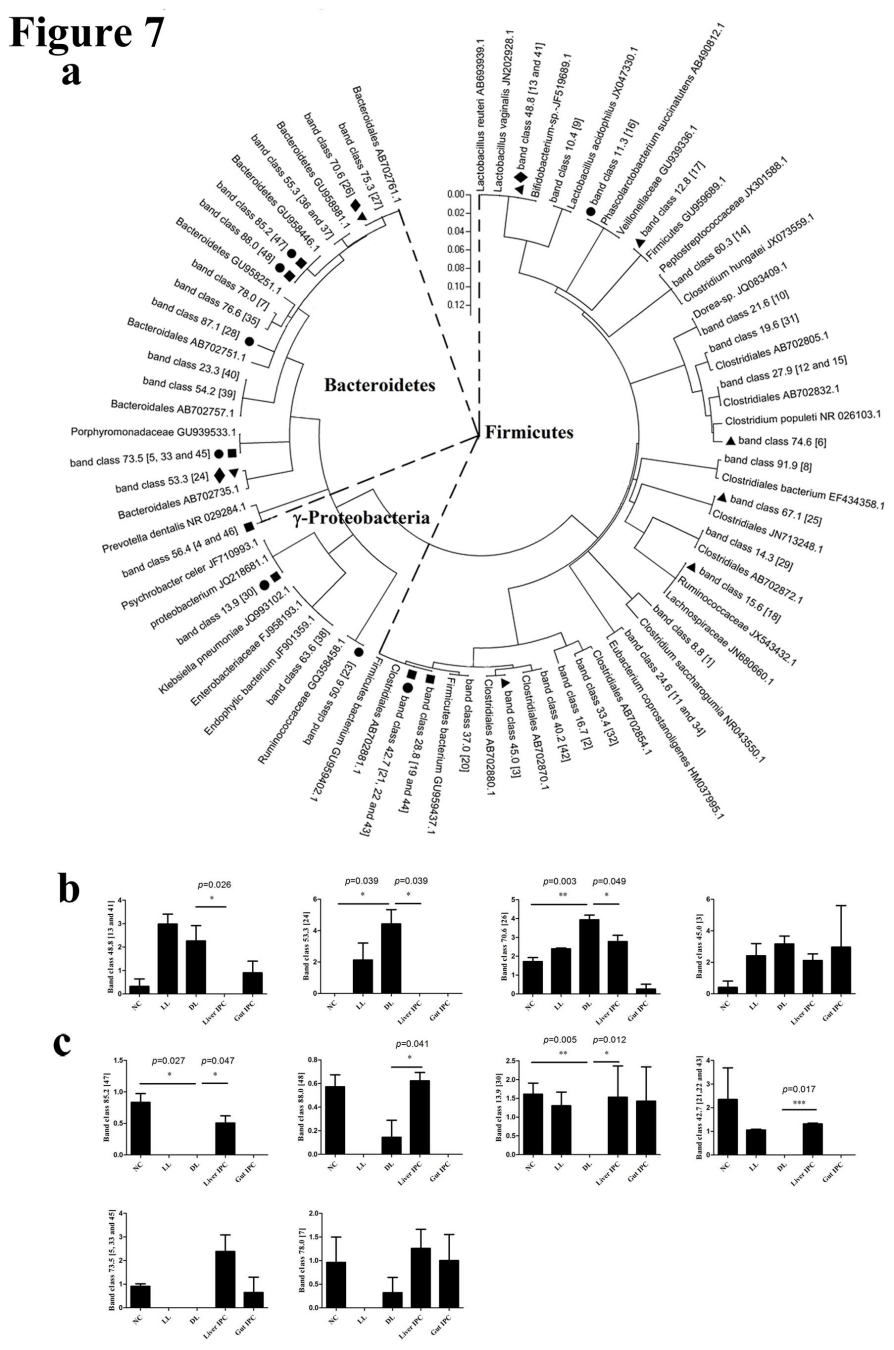
Phylogenetic tree of sequences using the neighbor-joining method (a) and key bands of structural shift of intestinal microbiota (b and c) from DGGE profiles. (a) Phylogenetic tree of sequences from DGGE profiles. The fragment sequences were named for their positions in gels using the band-matching tool with BioNumerics software version 6.01 (Applied Maths). The numbers shown in Figure 5 are in coincidence with the numbers shown in the brackets in the figure. 23 band classes without labels showed little variation in intensity among the different groups. Compared to the NC group, 8 band classes labeled with the *black* *square* showed a decrease in intensity, while 8 band classes labeled with the *black* *triangle* showed an increase in the DL group; Compared to the DL group, 3 band classes labeled with the *black* *diamond* showed a decrease in intensity, while 8 band classes labeled with the *black* *spot* showed an increase in the Liver IPC group. The plot was obtained from MEGA5 software (http://en.wikipedia.org/wiki/MEGA,_Molecular_Evolutionary_Genetics_Analysis). (b) The intensities of 3 key bands (band class 48.8, 53.3 and 70.6) were increased in the DL group and then decreased after liver IPC, and band class 45.0 also showed this trend. (c) The intensities of 4 key bands (band class 85.2, 88.0, 13.9 and 42.7) were decreased in the DL group and then increased after liver IPC. Both band classes 73.5 and 78.0 also showed this trend.

To clarify the phylogenetic relationship of intestinal bacterial species and find key bacteria of structural shift of intestinal microbiota induced by liver IPC following LT in rats, the phylogenetic tree of sequences from DGGE bands was done and analyzed (Figure 7). In the 48 PCR-DGGE bands analyzed in the study, 38 band classes were identified. DNA from at least 2 different samples were purified, sequenced and assigned to a bacterial species or phylotype based on the highest (90%-100%) sequence-similarity match to GenBank sequences obtained by BLAST analysis.

In Figure 7a, almost all matched bacteria of DGGE bands were assigned to 3 phyla: Firmicutes (63.3%), Bacteroidetes (27.8%) and gamma-proteobacteria (8.86%). Moreover, on the basis of gray amount of each band, we compared and analyzed change regulation of each band among the different groups. The results showed 23 band classes with little variation in band intensity among the different groups. The intensities of band classes 48.8% (band 13 and 41), 53.3% (band 24) and 70.6% (band 26) were significantly increased in the DL group versus the NC group, and decreased to the normal level in the Liver IPC group versus the DL group (Figure 7b). The band class 45.0% (band 3) had also a similar change trend. Meanwhile, The intensities of band classes 85.2% (band 47), 88.0% (band 48), 13.9% (band 30) and 42.7% (band 21, 22 and 43) were much lower in the DL group than the NC group, and higher in the Liver IPC group than the DL group (Figure 7c). The band classes 73.5% (band 5, 33 and 45) and 78.0% (band 7) also presented a similar change trend. The closest matched bacterial species corresponding to the above 10 key band classes of microbial structural shift could be found in the phylogenetic tree, as shown in Table **2**. In these key bacteria, 60% (6/10) were assigned to Bacteroidetes phylum, 30% (3/10) belonged to Firmicutes phylum and only 10% (1/10) were assigned to gamma-proteobacteria phylum.

**Table 2:** Identification of key bacteria of intestinal microbial structure shift.

*Band*	*Band class (%)*	*Closest BLAST match*	*Identity (%)*	*Accession no.*	*Phylum*	*DL vs. NC*	*Liver IPC vs. DL*
13, 41	48.8	*Lactobacillus reuteri Lactobacillus vaginalis* Bifidobacterium sp.	99	AB693939.1 JN202928.1 JF519689.1	Firmicutes	**↑**	**↓**
24	53.3	Bacteroidales	99	AB702735.1	Bacteroidetes	**↑**	**↓**
26	70.6	Bacteroidales	93	AB702761.1	Bacteroidetes	**↑**	**↓**
3	45.0	Clostridiales	93	AB702880.1	Firmicutes	**↑**	**↓**
47	85.2	Bacteroidetes	95	GU958446.1	Bacteroidetes	**↓**	**↑**
48	88.0	Bacteroidetes	95	GU958446.1	Bacteroidetes	**↓**	**↑**
30	13.9	Proteobacterium *Psychrobacter celer*	99	JQ218681.1 JF710993.1	gamma- Proteobacteria	**↓**	**↑**
21, 22, 43	42.7	Clostridiales Firmicutes bacterium	92	AB702881. 1GU959402.1	Firmicutes	**↓**	**↑**
5, 33, 45	73.5	Porphyromonadaceae	99	GU939533.1	Bacteroidetes	**↓**	**↑**
7	78.0	Bacteroidetes	92	GU958251.1	Bacteroidetes	**↓**	**↑**

## Discussion

Although the successful rate of LT has improved dramatically, early graft dysfunction remains a serious concern and also a main cause of higher morbidity after liver transplantation [24]. During LT, ischemia and subsequent reperfusion are the leading cause of early graft dysfunction in the current practice of organ transplantation [25]. From the first study of liver IPC reported by Toledo-Pereyra et al in a warm ischemic model [26], clinical trials and animal models sequentially proved the protective effect of IPC on liver I/R injury in surgery [27–29]. Recent studies have demonstrated that intestinal or hepatic I/R injury in surgery or organ transplantation would result in intestinal flora dysbiosis that follows epithelia damage [11–13].

However, so far the influence of IPC on intestinal microbiota has not been elucidated. In this study, we observed protective effects of liver IPC on hepatic graft, revealed beneficial roles of liver IPC on intestinal barrier function, analyzed changes of intestinal microbiota, and identified the key bacterial species of microbial structure shift related to liver IPC following LT in rats.

The intimate anatomical and functional relationship between the intestine and the liver closely links the two organs in health and disease. Hepatic injury or disease generally follows changes in intestinal permeability and microbial composition [14]. In patients with liver cirrhosis, community-wide changes of fecal microbiota mainly presented the prevalence of potentially pathogenic bacteria, such as Enterobacteriaceae and Streptococcaceae, with the reduction of beneficial populations such as Lachnospiraceae, which may affect patients’ prognosis [30]. Moreover, Hepatic I/R injury generally accompanies imbalance of intestinal microbiota, expressed by the increased Enterobacteria as well as the decreased Lactobacilli and Bifidobacteria [13]. Thus, liver function plays a critical role in maintaining intestinal microbial balance and stability.

Our results showed significantly ameliorated hepatic graft injury, improved intestinal barrier function, and partial restorations of intestinal microbiota in the Liver IPC group. Moreover, liver IPC increased intestinal microbial diversity and species richness, which presented a similar change profiling to normal control samples. Also, the cluster analysis showed a high similarity of DGGE profiling between liver IPC pretreatment and normal control, which was then confirmed by MDS and PCA. Furthermore, key bacteria corresponding to 10 key band classes of microbial structural shift induced by liver IPC were identified, and most of them were assigned to phylum Bacteroidetes. Collectively, these changes after liver IPC pretreatment were helpful for the stability and balance of intestinal microbiota, strongly proving the beneficial effect of liver IPC on intestinal microbiota in LT, which was speculated to be dependent on improved liver function by the “gut-liver axis”.

Interestingly, we found that gut IPC had little benefit on intestinal barrier function and microbial stability in a LT model except that gut IPC mildly attenuated intestinal mucosal epithelium integrity. The possible reason was that gut IPC only mildly increased the tolerance of intestinal epithelial cells to I/R injury, but had little influence on hepatic graft, thus it could not prevent microbial disturbances and cascade inflammation induced by the injured graft function. This result further proved the importance of liver function on intestinal microbial balance.

Under disease situation, intestinal microbiota imbalance can aggravate liver injury and even lead to severe liver disease. On one hand, intestinal bacterial products including LPS, flagellin, peptidoglycan and bacterial DNA activate the innate immune system to drive pro-inflammatory gene expression thus promoting chronic inflammatory disease of the liver [14]. For instance, intestinal microbial disruption could induce chronic liver inflammation and injury via Toll-like receptors pathway, ultimately promoting hepatocellular carcinoma (HCC) [31]. On the other hand, intestinal microbiota alter energy harvest and/or directly producing toxic metabolites playing a role in liver disease [14]. For example, the increase of deoxycholic acid as a gut bacterial metabolite known to cause DNA damage, can provoke senescence-associated secretory phenotype in hepatic stellate cells, which in turn secretes various inflammatory and tumour-promoting factors in the liver, thus facilitating HCC development in mice [32].

Due to the “gut-liver axis”, intestinal microbial restoration in turn is beneficial to recover liver function [33]. The cross-interactions of both further improve early hepatic graft dysfunction in LT, which may be a novel mechanism of liver IPC improving hepatic graft function in LT. Thus, we speculates that liver IPC can effectively improve hepatic graft function in LT, not only through directly regulating endothelial function, blood flow and neutrophilic activity to induce liver ischemic tolerance [34], but also through improving hepatic graft function to restore microbial balance, thereby further benefiting graft function by positive feedback of the “gut-liver axis”.

Intestinal microbiota is a complex ecological structure with an extensive microbial population [5,8]. The structure shift of intestinal microbiota always contributes or attributes to liver injury or its recovery in hepatic surgery [21]. Thus the unique feature of intestinal microbiota could be developed as a potential diagnostic biomarker predicting the severity of liver injury, as microbiota profiling may become a potential diagnostic biomarker of rejection in small bowel transplantation [35].

In conclusion, our study showed that liver IPC could not only improve hepatic graft function and intestinal barrier function, but also promote restorations of intestinal microbiota following LT, which may further benefit hepatic graft by positive feedback of the “gut-liver axis”.

## Supporting Information

Text S1 **Supplementary Material and Methods, including Experimental Design (Animals), Liver Function Detection (Liver H&E staining, Liver enzymes detection), Intestinal Barrier Function Determination (Intestinal ultrastructure observation, Serum endotoxin measurement, Serum TNF-α detection, Ileocecal sIgA detection, Bacterial culture in blood sample), and Microbial Analysis of the Ileocecal Content (DNA extraction, Quantitative PCR to detect bacterial population, DGGE profiling, Digital processing of DGGE profiles, Comparative analyses of DGGE profiles, Sequencing of DGGE bands) (DOC).**
(DOC)

Table S1 **Primers used in the study (DOC).**
(DOC)

Table S2 **Positive rate of bacterial culture in the blood (DOC).**
(DOC)
