# Supplementary material for: Liver Ischemic Preconditioning (IPC) Improves Intestinal Microbiota Following Liver Transplantation in Rats through 16s rDNA-Based Analysis of Microbial Structure Shift
Source: PLoS One. 2013 Oct 2;8(10):e75950. doi: 10.1371/journal.pone.0075950 (PMC3788797; doi:10.1371/journal.pone.0075950)
Supplement: Text S1 — Supplementary Material and Methods, including Experimental Design (Animals), Liver Function Detection (Liver H&E staining, Liver enzymes detection), Intestinal Barrier Function Determination (Intestinal ultrastructure observation, Serum endotoxin measurement, Serum TNF-α detection, Ileocecal sIgA detection, Bacterial culture in blood sample), and Microbial Analysis of the Ileocecal Content (DNA extraction, Quantitative PCR to detect bacterial population, DGGE profiling, Digital processing of DGGE profiles, Comparative analyses of DGGE profiles, Sequencing of DGGE bands) (DOC). (DOC) [file pone.0075950.s001.doc]

**MATERIALS AND METHODS**

***Experimental design***

**Animals** Specific pathogen-free (SPF) male inbred Lewis and DA rats (weight 220-250 g, 12-15 wk) were purchased from Beijing Vital River Laboratories (Beijing, China). All rats were housed in the Laboratory Animals Center of First Affiliated Hospital, School of Medicine, Zhejiang University. The rats were caged in 21°C, 12 h light/dark cycle, and fed with sterilized standard rat chow and water.

**Experimental protocol**

**Surgical procedures**

**Sample collection**

***Liver function detection***

**Liver H&E staining** The graft sample was fixed in 40 g/L neutral formaldehyde and embedded in paraffin, cut into 4 μm slices, stained with hematoxylin and eosin (H&E), and then analyzed blindly under light microscopy by a pathologist. The severity of I/R injury was graded by Suzuki’s criteria [1,2], with modifications. In this grading scheme, sinusoidal congestion, hepatocyte necrosis and ballooning degeneration are graded from 0 to 4. No necrosis, congestion/centrilobular ballooning is given a score of 0, while severe congestion/ballooning degeneration and more than 60% lobular necrosis is given a value of 4.

**Liver enzymes detection** Alanine aminotransferase (ALT) and aspartate aminotransferase (AST) of serum samples were detected by an automatic biochemical analyzer (Hitachi 7600, Tokyo, Japan).

***Intestinal barrier function determination***

**Intestinal ultrastructure observation** Ileal sample at 5 cm far from ileocecus was fixed into 2.5% glutaraldehyde (4°C, pH 7.4) and prepared for transmission electron microscopy (TEM) by the standard technical procedures. Intestinal mucosal ultrastructure was observed by TEM in Imaging Facility of Core Facilities, Zhejiang University School of Medicine, as previously described [3].

**Serum endotoxin measurement** Blood sample was placed in a pyrogen-free heparin-containing tube and centrifuged at 3000×g for 15 min at 4°C. 100 μL of serum was used for endotoxin measurement with the assay of Colorimetric Analysis for Limulus Test (Shanghai Yihua Medical Technology Co., Ltd, China) according to the manufacturer's instructions.

**Serum TNF-α detection** Serum TNF-α was assessed with enzyme-linked immunosorbent assay (ELISA) (Groundwork Biotechnology Diagnosticate Ltd., USA) according to the manufacturer's protocol.

**Ileocecal sIgA detection** Ileocecum contents (0.5 g) were homogenized in 1 mL PBS (pH 7.4) and centrifuged at 12 000×g for 20 min. The supernatant was taken for the measurement of sIgA by ELISA kit (RnD Ltd., USA) following the manufacturer's instructions. The sIgA level was presented as micrograms per gram of feces (μg/g).

**Bacterial culture in blood sample** Bacterial culture in the blood sample was performed by an automatic analyzer of bacteria (Model Viger 60, France) to identify the positive or negative results from the different samples.

***Microbial analysis of the ileocecal content***

**DNA extraction** DNA extraction and bacterial precipitation from the ileocecal feces were carried out using the Qiagen Stool Kit (QIAGEN, Hilden, Germany) with a modified protocol for cell lysis [4]. DNA integrity was checked by agarose gel electrophoresis and UV-light photography with ethidium bromide staining.

**Quantitative PCR (qPCR) to detect bacterial population** The primers for the genetic determinants were used in earlier studies **(Supplementary Table S1)**. All oligonucleotide primers were synthesized by TAKARA (Dalian, China). The qPCR was performed using a DNA Engine Opticon® 2 apparatus (Bio-Rad, California, US) related with the Opticon Monitor TM software (version 3.0; Bio-Rad, California, US) as our previous research [5]. A 20 µl amplification reaction was carried out with 10 µl SYBR® Green PCR Master Mix (ABI, Warrington, UK), mixed with the selected primer pairs at a concentration of 300 nM for each primer, and 1µl of the respective crude template DNA or water (negative control). Each reaction was done in duplicate and, _△_C(t) <0.5 is requisite. Amplifications were finished with the following profiles: one cycle at 95°C for 7 min, 40 cycles of denaturation at 95°C for 30 sec, annealing for 40 sec, 72°C for 30 sec, and fluorescence was measured after the extension phase of each cycle at an appropriate temperature for 10 sec. A final extension step at 72°C was sustained for 5 min. The annealing and plate-reading temperatures for each pair of primers are shown in Supplementary Table S1. The copy number of 16s rRNA operons per microliter of crude DNA template was determined by comparison with serially diluting plasmid DNA standards running on the same plate. The plasmid DNA standards were made from known concentrations of plasmid DNA that contained the respective amplicon for each set of primers, according to Bartosch *et al* [6]. The protocol to determine the detection limit of the assays was preformed as described previously [7].

**DGGE profiling** The V3 variable region of 16S rDNA was amplified by the hot-start touchdown protocol using specific primers for conserved regions of 16S rDNA [8]. The reaction mixture contained 2 µl of genomic DNA, 25 pmol of each primer, 4 µl of dNTP mixture, 5 µl of 10× Ex Taq buffer, and 0.5 µl of TaKaRa Ex Taq polymerase (TaKaRa, Dalian, China). The final volume was adjusted to 50 µl with sterile deionized water. The profiles were done in TProfessional Thermocycler (Biometra, Göttingen，Germany). 5 μl of the amplified products (approximately 200 bp) were checked by electrophoresis on 1% (wt/vol) agarose gel and visualized by ethidium bromide staining, and the concentrations were measured by using a NanoDrop ND-1000 spectrophotometer (Thermo Electron Corporation). All amplified products were stored at -20°C before DGGE analysis.

DGGE was carried out using the D-Code universal mutation detection system apparatus (Bio-Rad, Hercules, CA) with 16 cm by 18 cm by 1.5 mm gels according to the manufacturer's protocol. The sequence-specific separation of the PCR fragments was obtained in 8% (wt/vol) polyacrylamide (acrylamide-N, N’ bisacrylamide; 37.5:1 [wt/vol]) gels in 1× TAE buffer (40 mM Tris, 20 mM glacial acetic acid, 1 mM EDTA, pH 8.0). The denaturing gels contained a 35% to 75% gradient of urea and formamide increasing in the direction of electrophoresis. A volume of 20 μl of PCR fragment was loaded into the gel. Electrophoresis was conducted at a constant voltage of 80 V and a temperature of 60°C for approximately 16 h. Following electrophoresis, the gel was stained by SYBR green I (Sigma-Aldrich, Castle Hill, Australia) and photographed. On each DGGE gel, a standard reference in the middle and two at both ends were used for digital gel normalization and comparison among gels.

**Digital processing of DGGE profiles** DGGE profiles were processed digitally using BioNumerics software version 6.01 (Applied Maths, St-Martens-Latem, Belgium) in multistep procedure following the manufacturer’s instructions. All profiles were compared using the band matching tool and uncertain bands were included in the position tolerance settings. Parameters of band-classes allocation were ascertained following the previous research [9]. Bands occupying the same position across different lanes were matched and identified as the same band type. The quantitative information of a given band per sample was calculated by the software of Gel-Pro analyzer 4 (Media Cybernetics, USA) and exported as a data matrix. Species richness was calculated as the total number of each sample lane’s bands. Diversity was calculated using Shannon’s diversity and evenness index with quantitative information by the Past software (<http://folk.uio.no/ohammer/past/>)[10].

**Comparative analyses of DGGE profiles** Cluster analysis of DGGE pattern profiles were performed with the unweighted pair-group method with arithmetic means (UPGMA) based on the Dice similarity coefficient (band based) or the Pearson correlation coefficient (curve based). Multidimensional scaling (MDS) and principal components analysis (PCA) were used following the instructions of BioNumerics software. MDS is an optimized three-dimensional (3-D) manifestation of the similarity matrix, and the Euclidean distance between two points represents the similarity between them, providing a convenient visual interpretation. PCA is another way to visualize relationships among lanes using the lanes data (band classes). PCA reorients the plot to maximize the variation among lanes along the first three principal components in the 3-D plot.

**Sequencing of DGGE bands**  The interested DGGE bands were excised and placed into a sterile Eppendorf tube. The DNA was eluted form the gel slice into 20 μl of sterile water overnight at 4°C. The resulting DNA solution was then amplified again with universal bacterial primers F357+GC clamp and R518 as described above for DGGE. Amplicons without GC clamps were purified with QIAquick PCR purification Kit (Qiagen, Hilden, Germany), then ligated with pGEM-T Easy Vector (Promega, Madison, Wis.), transformed into competent Escherichia coli DH5 cells. The positive clones were verified and sequenced using the Sanger’s method on an ABI 3730 automated sequencing system (Invitrogen, Shanghai, China). Homology searches of the GenBank DNA database were performed using the BLAST tool. Based on the BLAST results, reference sequences of phylogenetic neighbor species (up to 90% similarity) were included for constructing phylogenetic tree using the MEGA 5.0 program in the method of neighbor-joining based on evolutionary distances.

***Statistical analysis***

REFERENCE

1. Suzuki S, Toledo-Pereyra LH, Rodriguez FJ, Cejalvo D (1993) Neutrophil infiltration as an important factor in liver ischemia and reperfusion injury. Modulating effects of FK506 and cyclosporine. Transplantation 55: 1265-1272.

2. Shen XD, Ke B, Zhai Y, Gao F, Anselmo D, et al. (2003) Stat4 and Stat6 signaling in hepatic ischemia/reperfusion injury in mice: HO-1 dependence of Stat4 disruption-mediated cytoprotection. Hepatology 37: 296-303.

3. Jiang JW, Ren ZG, Chen LY, Jiang L, Xie HY, et al. (2011) Enteral supplementation with glycyl-glutamine improves intestinal barrier function after liver transplantation in rats. Hepatobiliary Pancreat Dis Int 10: 380-385.

4. Bartosch S, Fite A, Macfarlane GT, McMurdo ME (2004) Characterization of bacterial communities in feces from healthy elderly volunteers and hospitalized elderly patients by using real-time PCR and effects of antibiotic treatment on the fecal microbiota. Appl Environ Microbiol 70: 3575-3581.

5. Lu H, Wu Z, Xu W, Yang J, Chen Y, et al. (2011) Intestinal microbiota was assessed in cirrhotic patients with hepatitis B virus infection. Intestinal microbiota of HBV cirrhotic patients. Microb Ecol 61: 693-703.

6. Bartosch S, Woodmansey EJ, Paterson JC, McMurdo ME, Macfarlane GT (2005) Microbiological effects of consuming a synbiotic containing Bifidobacterium bifidum, Bifidobacterium lactis, and oligofructose in elderly persons, determined by real-time polymerase chain reaction and counting of viable bacteria. Clin Infect Dis 40: 28-37.

7. Haarman M, Knol J (2006) Quantitative real-time PCR analysis of fecal Lactobacillus species in infants receiving a prebiotic infant formula. Appl Environ Microbiol 72: 2359-2365.

8. Muyzer G, de Waal EC, Uitterlinden AG (1993) Profiling of complex microbial populations by denaturing gradient gel electrophoresis analysis of polymerase chain reaction-amplified genes coding for 16S rRNA. Appl Environ Microbiol 59: 695-700.

9. Joossens M, Huys G, Cnockaert M, De Preter V, Verbeke K, et al. (2011) Dysbiosis of the faecal microbiota in patients with Crohn's disease and their unaffected relatives. Gut 60: 631-637.

10. Wang F, Li Q, Wang C, Tang C, Li J (2012) Dynamic alteration of the colonic microbiota in intestinal ischemia-reperfusion injury. PLoS One 7: e42027.
