# Supplementary material for: Liver Ischemic Preconditioning (IPC) Improves Intestinal Microbiota Following Liver Transplantation in Rats through 16s rDNA-Based Analysis of Microbial Structure Shift
Source: PLoS One. 2013 Oct 2;8(10):e75950. doi: 10.1371/journal.pone.0075950 (PMC3788797; doi:10.1371/journal.pone.0075950)
Supplement: Table S1 — Primers used in the study (DOC). (DOC) [file pone.0075950.s002.doc]

**Supplementary Table S1 Primers used in the study**

| Target group | Sequence (5’-3’) | Annealing temperature (°C) | Reference |
| --- | --- | --- | --- |
| Faecalibacterium prausnitzii | GATGGCCTCGCGTCCGATTAG  CCGAAGACCTTCTTCCTCC | 58 | [1] |
| Clostridium clusters I^a^ | TACCHRAGGAGGAAGCCAC  GTTCTTCCTAATCTCTACGCAT | 63 | [2] |
| Clostridium clusters XI^a^ | ACGCTACTTGAGGAGGA  GAGCCGTAGCCTTTCACT | 58 | [2] |
| Clostridium cluster XIVab^a^ | GAWGAAGTATYTCGGTATGT  CTACGCWCCCTTTACAC | 54 | [2] |
| Clostridium.bolteae | CCTCTTGACCGGCGTGT  CAGGTAGAGCTGGGCACTCTAGG | 62 | [2] |
| Bacteroides | GAAGGTCCCCCACATTG  CAATCGGAGTTCTTCGTG | 56 | [1] |
| Bifidobacterium spp. | GGGTGGTAATGCCGGATG  TAAGCCATGGACTTTCACACC | 59 | [1] |
| Lactobacillus | AGCAGTAGGGAATCTTCCA  ATTYCACCGCTACACATG | 58 | [1] |
| Enterococcus | AACCTACCCATCAGAGGG  GACGTTCAGTTACTAACG | 57 | [1] |
| Enterobacteriaceae | CATTGACGTTACCCGCAGAAGAAGC  CTCTACGAGACTCAAGCTTGC | 63 | [1] |

^a^The phylogeny of the genus Clostridium is according to the reference by Collins [3]

1. Bartosch S, Fite A, Macfarlane GT, McMurdo ME (2004) Characterization of bacterial communities in feces from healthy elderly volunteers and hospitalized elderly patients by using realtime PCR and effects of antibiotic treatment on the fecal microbiota. Appl Environ Microbiol 70:3575–3581.

2. Song Y, Liu C, Finegold SM (2004) Real-time PCR quantitation of clostridia in feces of autistic children. Appl Environ Microbiol 70:6459–6465.

3. Collins MD, Lawson PA, Willems A, Cordoba JJ, Fernandez-Garayzabal J, Garcia P, Cai J, Hippe H, Farrow JA (1994) The phylogeny of the genus Clostridium: proposal of five new genera and eleven new species combinations. Int J Syst Bacteriol 44:812–826.
