# Supplementary material for: Liver Ischemic Preconditioning (IPC) Improves Intestinal Microbiota Following Liver Transplantation in Rats through 16s rDNA-Based Analysis of Microbial Structure Shift
Source: PLoS One. 2013 Oct 2;8(10):e75950. doi: 10.1371/journal.pone.0075950 (PMC3788797; doi:10.1371/journal.pone.0075950)
Supplement: Table S2 — Positive rate of bacterial culture in the blood (DOC). (DOC) [file pone.0075950.s003.doc]

**Supplementary Table S2 Positive rate of bacterial culture in the blood**

| Groups | NC group | LL group | DL group | Liver IPC group | Gut IPC group |
| --- | --- | --- | --- | --- | --- |
| Bacterial positive number | 0/8 | 4/6 | 3/6 | 2/6 | 3/6 |
| Bacterial positive rates | 0% | 66.7% | 50% | 33.3% | 50% |
